# Effectiveness of Myofascial Release (MFR) vs. High-Frequency Transcutaneous Electrical Nerve Stimulation (TENS) for Pain Relief and Functional Improvement in College Students With Trapezius Myalgia

**DOI:** 10.7759/cureus.29898

**Published:** 2022-10-04

**Authors:** Aditi Joshi, Swapna Jawade, Neha Chitale

**Affiliations:** 1 Physiotherapy, Ravi Nair Physiotherapy College, Datta Meghe Institute of Medical Sciences, Wardha, IND; 2 Musculoskeletal Physiotherapy, Ravi Nair Physiotherapy College, Datta Meghe Institute of Medical Sciences, Wardha, IND

**Keywords:** stretching exercises, neck disability index, trapezius myalgia, tens, myofascial release

## Abstract

The pain in the trapezius muscle is known as trapezius myalgia. The patient often complains of trapezius muscle soreness and tightness. Muscle soreness usually lasts for a few days, if not longer. This muscular rigidity and stiffness cause spasms in upper trapezius fibres, culminating in neck discomfort in the posterior side of the neck and shoulder area. This protocol was designed to describe the study to evaluate the effect of myofascial release (MFR) versus high-frequency transcutaneous electrical nerve stimulation (TENS) for pain relief and functional improvement in subjects with trapezius myalgia.

Methods: Forty-five subjects with trapezius myalgia meeting inclusion criteria were selected for the study. Two groups were created, with group A undergoing MFR therapy and group B undergoing high-frequency TENS. The procedure was completed within four weeks. Regular assessments were carried out in the first week and fourth week of the rehabilitation. Throughout the recovery process, we evaluated pain, functional improvement, and range of motion of the neck at regular intervals. The outcome measures taken were the Numerical Pain Rating Scale (NPRS) and Neck Disability Index (NDI).

Discussion: The intervention's efficacy was assessed by looking at pain alleviation using the outcome measures. The study's findings strongly supported the application of these approaches and modalities in the rehabilitation of myalgia patients.

## Introduction

Myalgia, i.e., muscle pain can occur in any muscle. Trapezius myalgia is severe pain in trapezius muscle. Patient experiences pain, stiffness, and tightness in the trapezius upper fibres. This condition is characterized by acute or chronic pain. Neck pain is one of the usual musculoskeletal disorders in today’s era. According to various research and studies, around 67% of the population worldwide suffers at least once in a lifetime. Such pain may gradually increase and then eventually fade away, but the recovery is not complete frequently [1]. Mechanical reasons are the most common cause of neck pain. Mechanical neck pain is characterized by localized and/or transferred pain, as well as tender points, and limited cervical range of motion. If there is a restricted range of motion of the cervical spine, it might lead to increased muscle fatigue of anterior scalene, sternocleidomastoid, and upper fibres of the trapezius muscle, which can also hamper the breathing pattern in some patients [2]. The presence of myofascial trigger points specifies this type of musculoskeletal pain. These points are usually irritable nodules with taut bands present within the skeletal muscle. These are identified by palpating the area of pain [3]. Many people even complain of soreness in neck muscles. These complaints are now common in the student population due to prolonged sitting in front of computers or screens [4].

According to research, it has been confirmed that the most common type of neck pain is due to tenderness of muscle, that is, myalgia. In general, prolonged static work position influences such musculoskeletal problems of the neck, resulting in a few pathological alterations in the upper trapezius. Women are more afflicted than men. Regional muscle pain causes myofascial trigger points, which are found in the muscle belly. On palpation, the patient complained of pain and stiffness [5]. In the past, several physiotherapy protocols have been created that include rest, heat, ultrasound therapy, stretching, and strengthening exercises. The restriction in movement of the neck triggers or aggravates the pain and eventually leads to muscle spasms; therefore, making it difficult to treat and get the results. Stimulation of mechanical, neurological, and psychophysiological adaptation, as interfaced through the myofascial system, is the goal of the myofascial release (MFR) method, which is a soft tissue mobilization treatment. It calms tense muscles while improving blood circulation and lymphatic drainage. It also alters the viscoelasticity of connective tissue and restores muscle alignment [5].

Transcutaneous electrical nerve stimulation (TENS) is a non-invasive method that generates a low-frequency alternating electrical current that blocks spinal cord gates and releases endorphins on a sensory level. It has been researched and used to relieve a variety of pains and symptoms. TENS has been shown in prior studies to successfully ease pain in a variety of musculoskeletal ailments, including arthritic, low back, neuropathic, and postoperative pain. TENS is more effective in reducing pain as it can directly block the C-fibers that carry pain [6]. Low-frequency TENS (<10Hz) focuses more on treating chronic pain, whereas high-frequency TENS (80-100Hz) is frequently availed in treating acute pain as it stimulates the A-beta fibres [7]. It's uncertain whether the treatment works because of the different working circumstances of the subjects in previous research [8]. Henceforth, the goal of the research was to understand the efficacy of MFR and high-frequency TENS for relieving pain and functional improvement in patients having trapezius myalgia. The main objectives of this study were to observe the impact of the myofascial release technique in college students with chronic trapezius myalgia, to see the effect of high-frequency TENS in college students with chronic trapezius myalgia in relieving the pain and achieving functional improvement, and to compare the effect of myofascial release technique and high-frequency TENS in pain relief and functional improvement in students.

## Materials and methods

The study was conducted in the musculoskeletal-physiotherapy outpatient department (OPD) of Ravi Nair Physiotherapy College, Wardha, India, after receiving an agreement from the institutional ethics committee of Datta Meghe Institute of Medical Sciences. The study design was a cross-sectional study enrolling 45 participants. The participants enrolled in the study were divided randomly in a 1:1 manner into the MFR group (group A) and high-frequency TENS group (group B), for four weeks each, as this was the decided treatment period. Before being included, the participants were educated about the study's goals and approaches, and they signed written informed permission forms. The study schedule of enrolment, intervention, and assessment was done as recommended by protocol. The following were the eligibility criteria for participants.

Inclusion and exclusion criteria 

All patients included were those who were willing to participate, were between the ages of 18 and 25, both males and females, complained of neck discomfort and stiffness, and reported tightness in the upper trapezius muscle. Those over the age of 25, suffering from structural abnormalities such as torticollis or scoliosis, having any wound over the neck area, having a history of any upper limb surgery in the previous one year, healing from fractures, or having any skin disease over their back, and those who were registered in another clinical trial were all excluded from the study.

Timeline

Since the study lasted six months and the intervention lasted four weeks, participants were largely enrolled during the first two to three months of the study, resulting in a successful four-week intervention. The assessment took place on the first day of the visit and again after the fourth week of intervention. The subjects were asked to come in for treatment five days a week for four weeks.

Recruitment

Prospective patients were invited to be referred to our outpatient department (OPD) by health care practitioners affiliated with the institute. The patients were evaluated for study eligibility systematically using inclusion and exclusion criteria. After enrolling in the trial, individuals were randomly allocated into two groups, A and B, and undertook a four-week rehabilitation program with periodic assessments. Before randomization, an informed patient agreement was obtained after discussing the study's purpose, technique, potential benefits, and post-intervention effects. The research coordinator and lead investigators supervised the randomization process. Participants were asked to choose any allocation from the envelope for group assignment. 

Study procedure

The subjects were divided into two different groups and their demographic data was collected. Each participant underwent measurement of cervical range of motion using goniometry before the treatment. In group A, participants underwent MFR technique for the trapezius muscle, 10 minutes per day, four days a week, for four weeks. Deep transverse friction was given first which was followed by stretching of the upper trapezius muscle, all together for around 10 minutes. It included the use of techniques such as thumb after thumb pattern, palm’s ulnar border, and forearm to glide medially towards the upper scapular region. It was followed by the application of a cold pack over the trapezius to reduce the chances of soreness of the muscle. In every session, this same procedure was followed to get the desired results. In group B, the participants received high-frequency TENS. The frequency ranged between 80Hz and 100Hz and electrodes were applied over the trapezius muscle in a linear pattern. The participants in this group underwent 10 minutes of application of TENS at an intensity tolerated by the patient and capable of inducing upper trapezius muscle contraction four days per week for four weeks. This application was followed in every session to achieve the desired results. 

Outcomes

Numerical Pain Rating Scale (NPRS) is a numeric version of Visual Analogue Scale (VAS) in which the subject chooses a number (0-10 integers) that best depicts the severity of the patient's pain. This 11-pointer scale runs from "no pain" to "worst pain imaginable," with 0 indicating no pain and 10 representing extreme. Neck Disability Index (NDI) is created to give information on how neck pain has restricted a patient’s ability to manage everyday life. The questionnaire has a total of 10 segments and the total score is 50. For each segment, the highest possible score is 5 and the lowest is 0, where 0 means 'no pain' and 5 is 'severe pain'. An increased score indicates more patient-rated disability.

Data collection and management

The data for the analysis came from a pre-made spreadsheet containing a variety of baseline attributes. Data from the study was saved in a secure database. Hard copies of evaluation forms, signed informed permission, and other non-electronic documents were safely preserved in the study setting. Under the supervision of the lead investigators, data administration, data collection, and reporting were carried out. The accuracy of the research reports was double-checked. After the study, the Microsoft Excel spreadsheet (Microsoft Corporation, Redmond, Washington, United States) was published and submitted to the statistician for the required analysis. A checklist was used to avoid data loss due to incorrect staff procedures. Statistical analysis was done using unpaired and paired sample t-tests using IBM SPSS Statistics for Windows (IBM Corp., Armonk, New York, United States).

## Results

**Figure 1:**
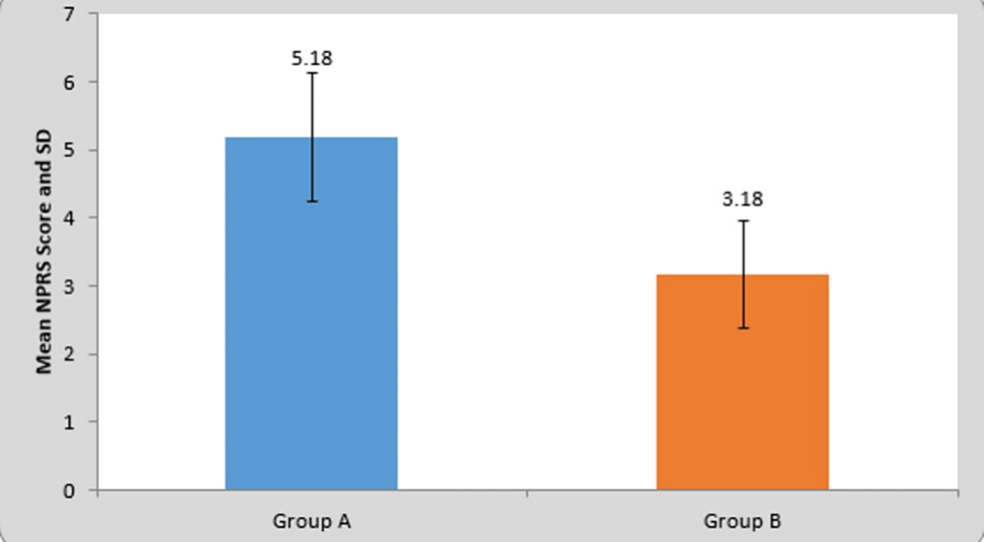
Comparison of mean difference in NPRS in groups A and B This graph depicts the mean NPRS score found in both groups after the intervention. It clearly shows that the intervention given in group A was more effective in decreasing the pain than the intervention given in group B. NPRS = Numerical Pain Rating Scale; SD = standard deviation

**Figure 2:**
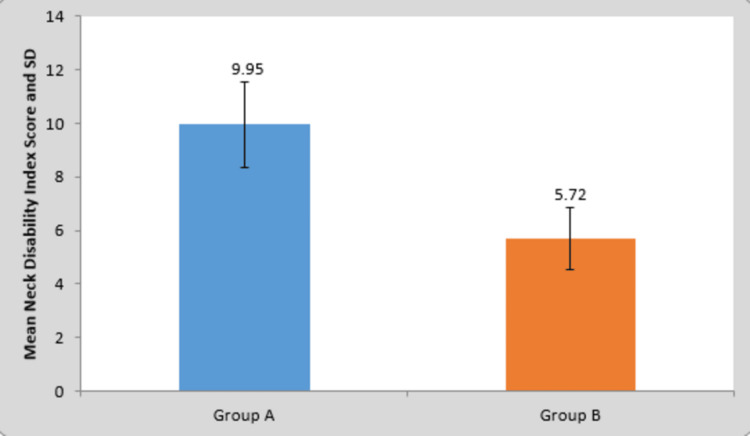
Comparing mean difference in the Neck Disability Index score in groups A and B This graph shows the mean Neck Disability Index score in group A and group B. The results were found to be much better in group A in comparison to group B. SD = standard deviation

**Figure 3:**
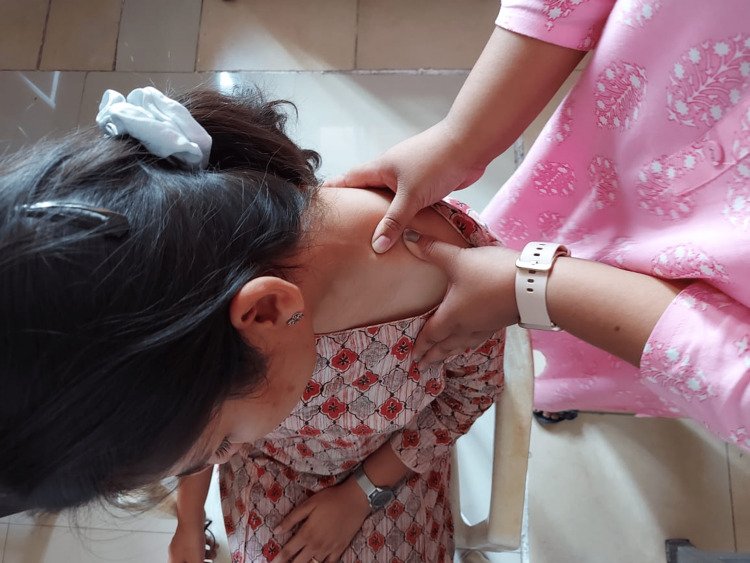
Myofascial release to upper fibres of trapezius muscle

**Figure 4:**
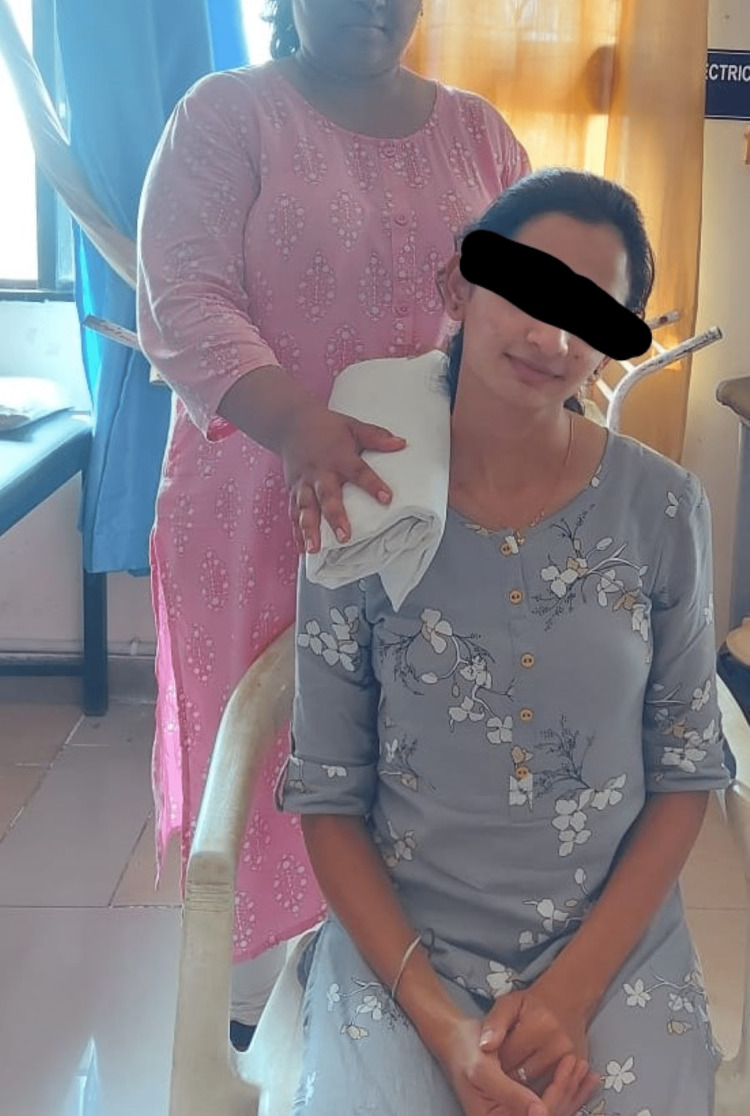
Cryotherapy for pain relief

**Figure 5:**
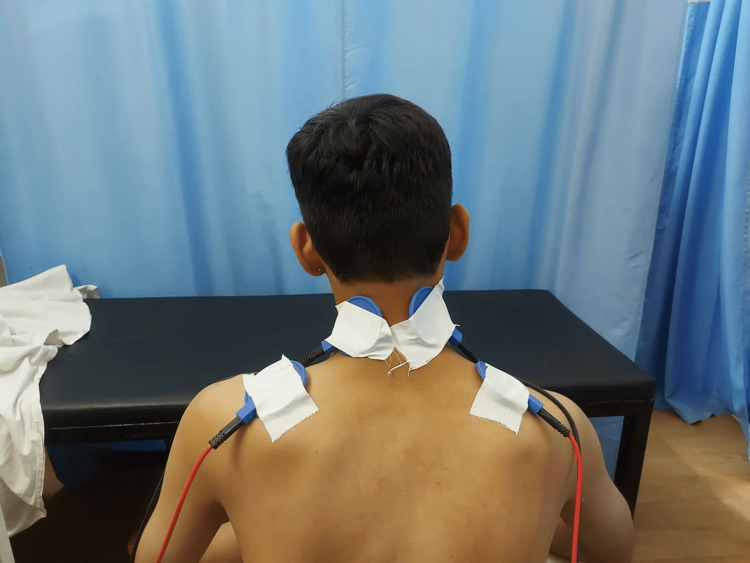
TENS for pain relief in the trapezius muscle TENS: Transcutaneous electrical nerve stimulation

The successful completion of this study provided the best treatment strategy in the care of patients with trapezius myalgia in order to give early pain relief and gain functional activities and functional range at the cervical joint. This study was useful in treatment in the healthcare environment, as it was indicated that both MFR and high-frequency TENS were helpful in alleviating the pain of trapezius muscle, but the MFR technique was more effective than high-frequency TENS. Statistical results showed the distribution of the patients in two groups was done according to their age. The age of the respondents in both groups differed just a little. The distribution of patients in two groups according to their gender was randomized, and it was noted that there were more females than males in both groups. Table 1 shows the difference between the two groups. The comparison between groups A and B for the NPRS can be seen in Figure 1, in the form of a graph. Table 2 depicts the mean difference in two groups for NDI and a graphical representation for the same can be seen in Figure 2. Figure 3, Figure 4, and Figure 5 show various treatment approaches given to patients, including MFR, cryotherapy, and TENS.

**Table 1:** Comparison of mean difference in NPRS in groups A and B This table depicts comparison of mean difference in NPRS in groups A and B. The values suggest the improvement in domain of pain after the intervention. NPRS = Numerical Pain Rating Scale

Group	N	Mean	Standard Deviation	Standard Error Mean	t-value
Group A	22	5.18	0.95	0.20	7.53 P=0.0001,S
Group B	22	3.18	0.79	0.16	

**Table 2:** Comparison of mean difference in the Neck Disability Index score in groups A and B This table depicts the comparison of mean difference in the Neck Disability Index score of group A and group B. It clearly shows that there was a more significant change in the functional improvement of the subjects in group A than in group B.

Group	N	Mean	Standard Deviation	Standard Error Mean	t-value
Group A	22	9.95	1.61	0.34	9.95 P=0.0001,S
Group B	22	5.72	1.16	0.24	

## Discussion

Neck pain is a significant health concern for many, affecting both function and well-being [7]. In trapezius myalgia, the patient complains of pain, stiffness, and tightness of the upper trapezius [9]. The purpose of this study was to evaluate and seek the effectiveness of MFR combined with high-frequency TENS for pain relief and functional improvement in individuals with trapezius myalgia. The primary goals were to examine the effect of the MFR technique on chronic neck pain in college students, to examine the effect of high-frequency TENS on pain relief and functional improvement in college students, and to compare the effects of the MFR technique and high-frequency TENS on pain relief and functional improvement in college students with chronic trapezius myalgia. MFR is a soft tissue mobilization technique that can be defined as the activation of mechanical, neurological, and psychophysiological adaptive capacity via the MFR. It works by relaxing tense muscles. In this study, two groups of students were selected who had complaints of severe neck pain for a prolonged period of time. This included 45 subjects, between the ages of 18 and 25. They were randomly distributed into two different groups to undergo different treatment protocols. Group A underwent MFR along with cryotherapy and stretching.

As per a study conducted by Chaudhary et al., MFR was more useful in lowering the pain threshold in upper trapezitis than cold pack by giving cryotherapy, but we found that when MFR is given as an intervention for chronic trapezius myalgia followed by cryotherapy, it gives good results rather than just applying a traditional intervention [7]. On the other hand, electrotherapy, including the TENS and interferential current (IFC), is one of the most often utilised physical therapy treatments in patients with neck pain. A study was conducted by Grabianska et al., and the objective was to examine the analgesic efficacy of TENS and IFC which would further help in reducing neck pain [10]. In 2018, Mishra et al. conducted an experiment to assess the impact of Active Release Therapy (ART) and MFR in treating trapezius muscular pain caused by spasm on 13 participants; that study found that ART helped to decrease pain fast and lengthen the fascia, but in our study, we found that applying MFR, the blood circulation of the local area increased and it lead to instant pain relief and was more comfortable for the patient while undergoing this treatment than any other, whereas a study conducted by Hattapoglu et al. stated that in chronic pain, electrotherapy modalities also helped in relieving pain and benefited the patients [6,11]. Another study conducted by Parab et al. described relieving upper trapezius spasm by the application of MFR and cryo-stretching. It proved that myofascial release can enhance range of motion as well as lead to immediate pain relief in comparison to cryo-stretching [12]. Battecha et al. in their study conducted on 28 females for myofascial pain syndrome stated that an amalgam of microcurrent therapy and traditional exercises was helpful in handling patients with myofascial discomfort [13].

TENS has been researched and used to relieve a variety of pains and symptoms. It is a non-invasive method that has been shown to effectively alleviate pain in cases of musculoskeletal, arthritic, low back, neuropathic, and postoperative pain. When we conducted this study, we found that when high-frequency TENS was applied to the affected area, the pain-relieving effect was faster than in low-frequency TENS. According to a few other studies, it has been documented that the usage of TENS also enhances motor activity. Battecha et al. also found that TENS did not play any significant role in decreasing pain in athletes with muscle soreness, whereas cryotherapy was quite effective in reducing pain [13]. But in our study, we found that when TENS was applied with high frequency, it gave quick and prolonged results for reducing chronic pain in patients with chronic trapezius myalgia. Gibson et al. in their study found that TENS also helped in relieving neuropathic pain, while Witkoś et al. found that application of TENS relaxed the muscles and therefore, even helped in reducing anxiety and depression in such patients. Witkos et al. in their study stated that the length of depression had no effect on the current-induced stimulus [14,15]. The study's purpose was to investigate and assess the effects of MFR and high-frequency TENS in participants with trapezius myalgia. The NPRS and NDI scales aided in determining the patient's pain severity and functional improvement.

## Conclusions

MFR is effective in patients suffering from chronic trapezius myalgia. The study found improvement in both groups when pain and functional mobility of the neck were assessed following the intervention period, utilizing the NPRS and NDI to score. However, the group that underwent MFR followed by application of a cold pack showed more significant results than the other group, which underwent a high-frequency TENS modality. Therefore, the individualized intervention of MFR with a cold pack is recommended for reducing pain and for functional improvement.
